# Ethical Gambling: A Necessary New Point of View of Gambling in Public Health Policies

**DOI:** 10.3389/fpubh.2018.00012

**Published:** 2018-01-31

**Authors:** Mariano Chóliz

**Affiliations:** ^1^Universitat de València, Valencia, Spain

**Keywords:** pathological gambling, public health, ethical gambling, gambling policies, gambling addiction prevention

“Corporate social responsibility” (1, 2) or “responsible gambling” (RG) (3, 4) are concepts that mediate the idea that gambling can be harmful to some gamblers, and the assumption that governments and companies can do something to mitigate the problems caused by gambling and to promote “appropriate” patterns of behavior in gamblers.

Although many governments and companies have implemented strategic plans based on models of RG, this has not served to prevent one of the major public health problems caused by gambling, which is gambling disorder. On the contrary, gambling has increased globally, the initiatives based on RG models have had little relation to the research evidence on best practices to prevent harms (5, 6) and the independent gambling researchers that could improve RG initiatives are often ignored by policy makers (7–9). For Livingstone and Woolley (10), the term is limited by its lack of clear goals and terminological clarity.

This paper defends the need for a specific concept: “ethical gambling” (EG), which goes one step further in assuming the responsibility of companies and governments not only with regard to the cause of the damage gambling induces but also with respect to the specific actions to prevent gambling disorder. The purpose of EG is to create the necessary environmental conditions that permit gambling as an economic activity, but with the primary objective of preventing potential health risks, primarily gambling disorder.

In order to be able to understand the concept EG, it is necessary to consider the following assumptions with regard to gambling (11):

(a) Regarding its socioeconomic dimensions:
  - Gambling is a major economic activity in societies where it is allowed. According to the Global Betting and Gaming Consultants (12), the global gambling revenue was estimated to be US $464 billion in 2016.
  - In terms of Game Theory (13), gambling is an example of a zero-sum game, a non-cooperative game in which one player’s wins equals the other player’s losses, resulting in a net benefit of 0. In general terms, gambling may be thought of as a zero-sum game in which the gambling company is one player and the gamblers constitute the other.
  - Gambling is designed in such a way that the expected value, also known as mathematical expectation, is favorable for the company that manages the game. Therefore, the more money spent on gambling, the greater the probability that the companies managing the games will win. That is, the more money played, the greater the profits obtained.
(b) From a psychological perspective:
  - Gambling is a potentially addictive activity because: (a) it activates the same reward circuits in the brain as drugs and (b) the clinical characteristics of gambling disorder are the same as those of drug addictions or alcoholism (14).
  - Gambling disorder is a serious mental disorder (15, 16), in which the clinical and diagnostic criteria point to a pattern of excessive gambling.
  - This pattern of excessive gambling places the gambler in a spiral of increasingly important losses, given that the mathematical expectation is not favorable to the gambler. One of the most characteristic symptoms of gambling addiction is that the gambler plays to try to recover losses (known as “chasing” one’s losses), which, of course, makes the problem worse (17).
  - Pathological gamblers continue to play despite awareness that doing so will seriously harm them and their families, or entail problems with the law because of using illegal means to obtain the money with which to keep gambling. This information on the negative effects of their pattern of excessive gambling is not enough to make them stop (18). Gambling addicts are not able to control their gambling behavior. In most cases, they find it very difficult to stop playing, given that the disorder has made them dependent on the game, as occurs with addicts to substances of abuse (19).

In short, gambling companies are businesses that benefit financially from gamblers’ losses. The benefits are greater the more the gamblers play because the mathematical expectation is in favor of the gambling companies. Therefore, it is in the interest of the companies to promote gambling through marketing and by ensuring that the games are easily accessible and readily available. The crucial issue is that a significant proportion of gambling revenue (between 15 and 40%) is derived from problem gamblers (20–22). The defining feature of a problem gambler is not only the emergence of negative consequences but also the presence of a subjective sense of impaired control (23). Self-report instruments can be used for evaluating problem gambling for research purposes as well as in clinical contexts (24–30).

With respect to the causes and the prevention of gambling disorder, two lines of evidence have been demonstrated. First, gambling disorder is an addictive disorder (14, 16) in which the majority of their clinical symptoms induce excessive gambling: (a) *tolerance*: the need to gamble with increasing sums of money to achieve the desired excitement; (b) the “*chase*”: after losing money, individuals often return to win back their losses; (c) *impaired control*: repeated unsuccessful attempts to control or stop gambling; (d) gambling when *feeling distressed* (i.e., helpless, guilty, anxious, or depressed); and (e) *emotional agitation* when gambling is interrupted.

Second, gambling policies are the main strategies for preventing excessive and gambling disorder (31). Some of these strategies are:

(a) Restrictions on the general availability of gambling. A meta-analysis of 34 studies made in Australia and New Zealand demonstrated the relationship between density of electronic gambling machines (EGMs) and gambling disorder (32). Other researchers in United States demonstrated the relationship between problem gambling and the existence of a casino within 10 miles of the gambler’s home (33, 34).
(b) Restricting more harmful types of gambling. Not all forms of gambling are equally problematic (35–40). EGMs are the form of gambling most often identified as creating the most problems in Japan (35), Germany (41), France (42), and in many other Western countries (36). Other forms of continuous gambling, as casino table games, are often demonstrated as the more problematic gambling in Asian countries (43, 44). Similarly to drugs, governments should include restrictions on the most addictive gambling. In general, the risks for problem gambling are higher in gamblers in which the consumption is centered on games which have more rapid event frequency (45).
(c) Player pre-commitment. Consists of putting limits on time, frequency, or money prior to the start of play. In spite of the controversial results obtained in some studies in Canada, Australia, or Norway (46), this has been due to methodological problems because of the many different ways it can be implemented (mandatory or voluntary; revocable limits or not, different duration of limits, etc.) (40). Pre-commitment holds promise as a harm-minimization technique (47, 48).

These facts highlight the need for the regulation of gambling and implementation of effective public health policies (49).

## Actions Proposed

The core assumption of EG is that the environmental conditions—which are the main factors that cause gambling disorder—can be best achieved by means of gambling regulation using the findings of evidence-based research. These actions are compatible and complementary to the implementation of prevention programs, similar to those that have been developed for the prevention of licit drugs (tobacco and alcohol) (50–52).

**Figure 1:**
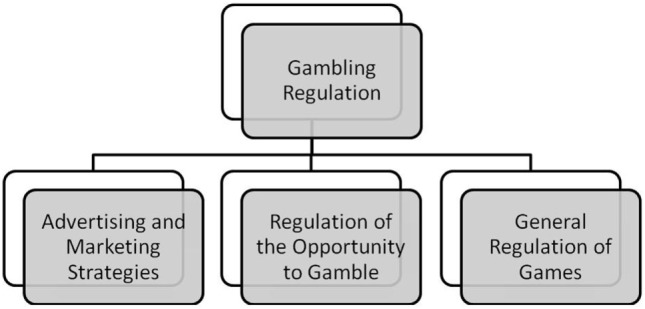
Gambling regulation based on ethical gambling assumptions.

The proposed regulation model [(53); Figure 1] has three dimensions: (a) gambling advertising and promotional strategies used by the gambling companies, (b) opportunity to gamble (availability and accessibility of games), and (c) the rules of the games themselves.

- Advertising and marketing. Prevention of gambling addiction is incompatible with the promotion of excessive gambling (54). Nevertheless, unlike tobacco and alcohol, which have restrictions on advertising, and there are campaigns to prevent their consumption (55–57), gambling is a widely promoted activity. For that reason, advertising and marketing techniques should be regulated through laws. Gambling regulation policies based on the principles of EG should present information about gambling in a truthful way, along with the consequences to one’s health and wellbeing, to avoid promoting games considered to be the most addictive, and to take the necessary measures to avoid advertising and promotion techniques in especially vulnerable communities.
  As a general strategy, gambling advertising should be limited to gambling venues: bingo halls, casinos, bookmaking and betting outlets, and gambling websites in the case of online gambling. Some kinds of marketing techniques, such as bonuses and loyalty cards, should be banned.
- Opportunity to gamble: The main objective is to reduce the availability and accessibility of gambling to prevent the emergence of gambling addiction due to the relationship of these factors to gambling behavior and pathology (34). This includes the following:
  *Strategies to reduce availability*: Limiting the number of casinos or game rooms and regulating the distances between gambling venues, authorizing gambling only in gaming rooms or casinos, and limiting online gambling (companies and type of bets).
  *Strategies to reduce accessibility*: Individuals must show reliable personal identification before being allowed to gamble (all types), implementation of an Interdictions General Registry by which gamblers can limit their access to games anywhere in the country, and the use of passwords for all electronic games and online gambling.
  However, the fact that many people gamble at non-problematic levels suggests that low-level exposure to gambling is not sufficient to induce gambling disorder in people with no pre-disposition to this syndrome. So, the threshold for refining gambling activities and policies around gambling should be set based on the likelihood that a vulnerable individual may begin to escalate their gambling behavior (i.e., start to transition to “addiction”). Therefore, an important practical addition to the EG strategy might be to pro-actively fund/target research into aspects of gambling that induce excessive/maladaptive responses in individuals at high risk for gambling disorder.
- Game rules: These measures must directly affect the rules of the games themselves and the conditions in which they are presented, because structural and situation factors associated with gambling contribute to persistence in play and the development of excessive and problem gambling behaviors (58). The government must establish a pool of specific measures, not only favoring an adaptive gambling pattern but also avoiding, as far as possible, the development of excessive and gambling disorder.
  To satisfy that objective, the structural characteristics of the most addictive games should be modified according the scientific research (38, 59, 60). This may include restrictions on gambling speed (61); delaying the time between the bet and the outcome (62); reduction of maximum bet size (63); diminishing the percentage of win; posting the payoff probabilities (average percent of spins that yield non-0 payoff); posting the ratio of amount won (on average) for every $1 wagered; minimizing sensory cues (bells, lights) that augment the salience of wins—and maximizing the sensory cues associated with losses (which are often absent); reducing the frequency of “near-miss” outcomes on EGMs; introduction of pre-commitment techniques whereby preset limits on time, frequency, or money spent are registered before playing begins, etc. Additional changes should be incorporated to remove or reduce factors that may promote chasing or otherwise aggravate or potentiate a pre-existing vulnerability to engage in excessive gambling.

The key measure proposed is a personal gambling smart card (PGSD) issued by the government that would be mandatory for all types of gambling. The PGSD would record all gambling wins and losses with the aim of preventing large daily, weekly, or monthly losses by blocking gambling for the rest of the day, week, or month when the gambler reaches the limit previously established by the government.

In sum, EG is not meant to replace other concepts, such as RG; however, a different point of view with regard to gambling is necessary in order to prevent gambling disorder. RG focuses on helping gamblers to be well-informed about the risks of gambling and the actions they can take to avoid addiction problems, as well as on how to create the appropriate conditions so that the gambler can play or stop playing freely (3).

The main problem with RG models is that they consider that gambler is the foremost answerable for their disorder, while placing little or no emphasis on harm-inducing gambling policies (6). Consequently, gambling industry implements predatory gambling practices (60) that contribute to gambling-related harm.

Ethical gambling also assumes the importance of information about the risks of gambling and the relevance of knowledge about the actions a player can take to avoid addiction. However, this is not sufficient because: (a) gambling is potentially addictive, (b) the conditions under which it occurs potentiate excessive gambling and the addictive effects of gambling, and (c) pathological gamblers cannot control gambling behavior by themselves. The main solution is that the administration sets in place effective measures to prevent gamblers playing excessively and, in particular, to prevent them from losing excessive amounts of money.

So, a different point of view with regard to gambling is necessary for several reasons. First, it is important to understand how gambling is organized in today’s society and the consequences it has on public health, specially on gambling disorder. Second, a better understanding of the consequences of gambling may facilitate the implementation of preventative regulations that might otherwise be misunderstood and generate controversy, such as limiting the losses of gamblers. Third, several gambling operators have obtained certificates for implementing RG measures because they recommend that customers gamble responsibly; however, at the same time, they use marketing strategies, such as bonuses and loyalty cards, that are particularly harmful for the most vulnerable gamblers, which misrepresents the meaning of RG.

Gambling policies must be adapted to the cultural and legal dictates of individual countries. Moreover, in democratic societies, principles such as those of EG should guide authorities to regulate gambling in a way that promotes healthy habits and prevents gambling disorder. Science should play a key role in gambling policies.

For gambling companies and stakeholders, gambling is an economic activity that produces significant economic benefits; therefore, we stipulate that the government, rather than such companies, impose gambling regulations because it is unlikely that gambling companies will voluntarily enforce effective regulatory measures. The profit margin of gambling operators is the gross gambling revenue (GGR), which is the amount of money gamblers have lost, but a significant proportion of GGR is derived from problem gamblers. According to Game Theory, gambling is a zero-sum game with the peculiarity that the outcome of the game always favors the one who manages the gambling. The only solution for the gambler is make the decision to stop gambling before losses become catastrophic or result in gambling disorder. However, because pathological and problematic gamblers are not able to rationally make that decision, and gambling operators have no interest in preventing their “best” customers from gambling, such decisions must be made by public authorities. The *sine qua non* condition is that public authorities uphold the right to health over the free market preventing the gambler from having large losses.

In short, EG addresses a highly controversial contemporary issue, the moral limits of the market, which was masterfully summarized by Michael Sandel when he noted that the problem with society today is that “we drifted from having a market economy to being a market society” [(64), p. 6].

## Author Contributions

MC is the main author. He has developed the concept and he has proposed to the Spanish authorities a gambling regulation model in order to prevent pathological gambling.

## Conflict of Interest Statement

The author declares that the research was conducted in the absence of any commercial or financial relationships that could be construed as a potential conflict of interest.
